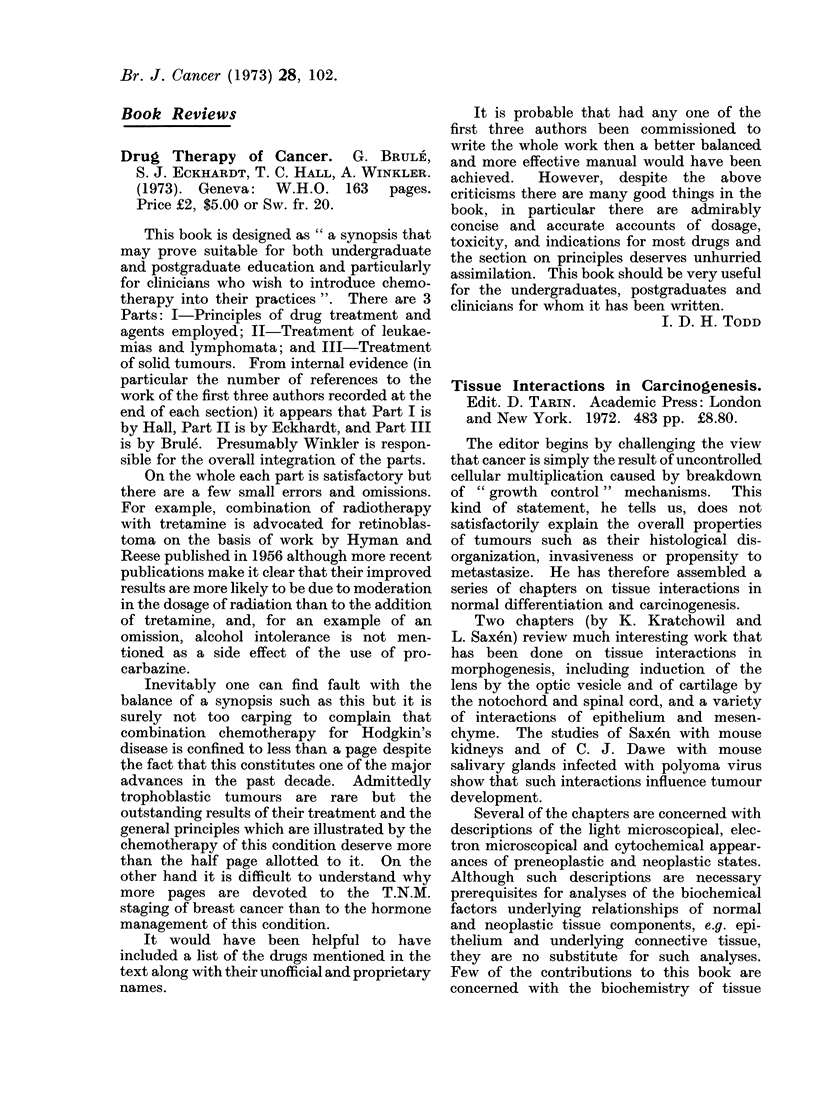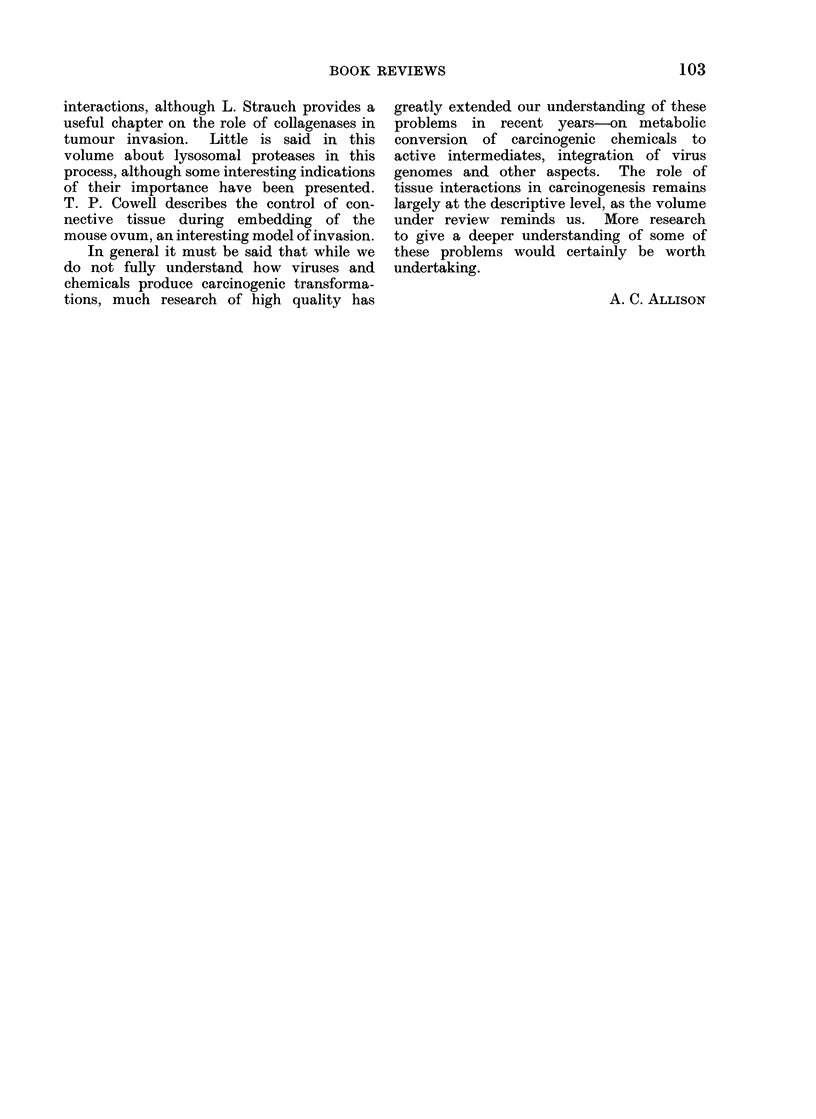# Tissue Interactions in Carcinogenesis

**Published:** 1973-07

**Authors:** A. C. Allison


**Tissue Interactions in Carcinogenesis.** Edit. D. TARIN. Academic Press: London and New York. 1972. 483 pp. £8.80.

The editor begins by challenging the view that cancer is simply the result of uncontrolled cellular multiplication caused by breakdown of " growth control " mechanisms. This kind of statement, he tells us, does not satisfactorily explain the overall properties of tumours such as their histological disorganization, invasiveness or propensity to metastasize. He has therefore assembled a series of chapters on tissue interactions in normal differentiation and carcinogenesis.

Two chapters (by K. Kratchowil and L. Saxén) review much interesting work that has been done on tissue interactions in morphogenesis, including induction of the lens by the optic vesicle and of cartilage by the notochord and spinal cord, and a variety of interactions of epithelium and mesenchyme. The studies of Saxén with mouse kidneys and of C. J. Dawe with mouse salivary glands infected with polyoma virus show that such interactions influence tumour development.

Several of the chapters are concerned with descriptions of the light microscopical, electron microscopical and cytochemical appearances of preneoplastic and neoplastic states. Although such descriptions are necessary prerequisites for analyses of the biochemical factors underlying relationships of normal and neoplastic tissue components, *e.g.* epithelium and underlying connective tissue, they are no substitute for such analyses. Few of the contributions to this book are concerned with the biochemistry of tissue interactions, although L. Strauch provides a useful chapter on the role of collagenases in tumour invasion. Little is said in this volume about lysosomal proteases in this process, although some interesting indications of their importance have been presented. T. P. Cowell describes the control of connective tissue during embedding of the mouse ovum, an interesting model of invasion.

In general it must be said that while we do not fully understand how viruses and chemicals produce carcinogenic transformations, much research of high quality has greatly extended our understanding of these problems in recent years—on metabolic conversion of carcinogenic chemicals to active intermediates, integration of virus genomes and other aspects. The role of tissue interactions in carcinogenesis remains largely at the descriptive level, as the volume under review reminds us. More research to give a deeper understanding of some of these problems would certainly be worth undertaking.

A. C. Allison